# Chemoembolization combined radiofrequency ablation vs. chemoembolization alone for treatment of beyond the Milan criteria viable hepatocellular carcinoma (CERFA): study protocol for a randomized controlled trial

**DOI:** 10.1186/s13063-023-07266-4

**Published:** 2023-03-28

**Authors:** Soon Kyu Lee, Hyun Yang, Jung Hyun Kwon, Dong Jae Shim, Doyoung Kim, Soon Woo Nam, Sun Hong Yoo, Si Hyun Bae, Ahlim Lee, Young Joon Lee, Changho Jeon, Jeong Won Jang, Pil Soo Sung, Ho Jong Chun, Su Ho Kim, Joon-Il Choi, Jung Suk Oh, Yun-Jung Yang

**Affiliations:** 1 Department of Internal Medicine, Incheon St. Mary’s Hospital, College of Medicine, The Catholic University of Korea, Seoul, South Korea; 2 Department of Internal Medicine, Eunpyeong St. Mary’s Hospital, College of Medicine, The Catholic University of Korea, Seoul, South Korea; 3 Department of Radiology, Incheon St. Mary’s Hospital, College of Medicine, The Catholic University of Korea, Seoul, South Korea; 4 Department of Radiology, Eunpyeong St. Mary’s Hospital, College of Medicine, The Catholic University of Korea, Seoul, South Korea; 5 Department of Internal Medicine, Seoul St. Mary’s Hospital, College of Medicine, The Catholic University of Korea, Seoul, South Korea; 6 Department of Radiology, Seoul St. Mary’s Hospital, College of Medicine, The Catholic University of Korea, Seoul, South Korea; 7 Institute of Biomedical Science, Catholic Kwandong University International St. Mary’s Hospital, Incheon, South Korea

**Keywords:** Hepatocellular carcinoma, Radiofrequency ablation, Transcatheter arterial chemoembolization, Randomized controlled trial

## Abstract

**Background:**

Many previous studies evaluated a combination of transcatheter arterial chemoembolization (TACE) and radiofrequency ablation (RFA) for treating early hepatocellular carcinoma (HCC); however, studies evaluating combination therapy for beyond-the-Milan criteria HCC are scarce.

**Methods:**

A total of 120 patients with beyond-the-Milan criteria HCC who have viable tumour after first TACE will be enrolled in this multi-institutional, parallel, pragmatic, randomized controlled trial. Patients with metastasis, vascular invasion, or a sum of tumour diameter > 8 cm will be excluded. Eligible patients will be randomly assigned to combination TACE and RFA therapy or TACE monotherapy groups. Patients in the combination therapy group will receive a second TACE and subsequent RFA at the viable tumour. Patients in the TACE monotherapy group will receive only second TACE. Patients in both groups will undergo magnetic resonance imaging 4–6 weeks after second TACE. The primary endpoint is 1-month tumour response, and secondary endpoints are progression-free survival, overall response rate, number of treatments until CR, overall survival, and change in liver function.

**Discussion:**

Although TACE can be used to treat intermediate-stage HCC, it is difficult to achieve CR by first TACE in most intermediate-stage patients. Recent studies show a survival advantage of combination therapy over monotherapy. However, most studies evaluating combination therapy included patients with a single tumour sized < 5 cm, and no studies included patients with intermediate-stage but more advanced (i.e., beyond-the-Milan criteria) HCC. This study will evaluate the efficacy of combined TACE and RFA therapy for patients with advanced HCC within the intermediate stage.

**Trial registration:**

Clinical Research Information Service (CRiS) KCT0006483.

## Administrative information

Note: the numbers in curly brackets in this protocol refer to SPIRIT checklist item numbers. The order of items has been modified to group similar items (see http://www.equator-network.org/reporting-guidelines/spirit-2013-statement-defining-standard-protocol-items-for-clinical-trials/).

**Table:** 

Title {1}	Chemoembolization Combined Radiofrequency Ablation vs. Chemoembolization Alone for Treatment of beyond the Milan Criteria viable Hepatocellular Carcinoma (CERFA): Study protocol for a randomized controlled trial
Trial registration {2a and 2b}	Registry: Clinical Research Information Service (CRiS) (https://cris.nih.go.kr/cris) Identifier: KCT0006483 (https://cris.nih.go.kr/cris/search/detailSearch.do?seq=20870&status=5&seq_group=20174&search_page=M) Date of Registry: August 20, 2021
Protocol version {3}	Version 2.1, November 15, 2022
Funding {4}	1.The National Research Foundation of Korea (NRF) grant funded by the Korean government (MSIT, no. NRF-2021R1G1A1010823) 2.The Korean Society of Radiology through Radiology Imaging Network of Korea for Clinical Research (RINK-CR-2021–002) 3.STARmed Co., Ltd., Goyang-si, Gyeonggi-do, Korea 4.A Radiological Research Fund of Department of Radiology, The Catholic University of Korea for 2021
Author details {5a}	^1^Soon Kyu Lee, ^2^Hyun Yang, ^1^Jung Hyun Kwon*, ^3^Dong Jae Shim*, ^3^Doyoung Kim, ^1^Soon Woo Nam, ^1^Sun Hong Yoo, ^2^Si Hyun Bae, ^2^Ahlim Lee, ^4^Young Joon Lee, ^4^Changho Jeon, ^5^Jeong Won Jang, ^5^Pil Soo Sung, ^6^Ho Jong Chun, ^6^Su Ho Kim, ^6^Joon-Il Choi, ^6^Jung Suk Oh, ^7^Yun-Jung Yang Department of ^1^Internal Medicine and ^3^Radiology, Incheon St. Mary's Hospital, College of Medicine, The Catholic University of Korea, Seoul, Korea Department of ^2^Internal Medicine and ^4^Radiology, Eunpyeong St. Mary's Hospital, College of Medicine, The Catholic University of Korea, Seoul, Korea Department of ^5^Internal Medicine and ^6^Radiology, Seoul St. Mary's Hospital, College of Medicine, The Catholic University of Korea, Seoul, Korea ^7^Institute of Biomedical Science, Catholic Kwandong University International St. Mary’s Hospital, Incheon, Korea SKL and HY contributed equally to this work and are co-first authors. ^*^Correspondence to Dong Jae Shim or Jung Hyun Kwon
Name and contact information for the trial sponsor {5b}	Dong Jae Shim, MD, PhD inharad@naver.com 56 Dongsu-ro, Bupyeong-gu, Incheon, Republic of Korea, 21431 Department of Radiology, Incheon St. Mary's Hospital, College of Medicine, The Catholic University of Korea
Role of sponsor {5c}	All sponsors supported this study through grants. None of the funders had roles in the study design, data collection and analysis, decision to publish, or preparation of the manuscript.

## Introduction

### Background and rationale {6a}

Hepatocellular carcinoma (HCC) is the fifth most common cancer and the second leading cause of cancer-related mortality worldwide [1]. Although active surveillance programs and the widespread use of imaging have improved its early detection, HCC is often found at an advanced stage, and the outcomes of both local and systemic treatments are largely unsatisfactory. According to Barcelona Clinic Liver Cancer staging, the recommended treatment for HCC of an intermediate stage (B) is transcatheter arterial chemoembolization (TACE) [2]. However, the intermediate stage encompasses large heterogeneity in tumour size, number, location, and hepatic function. Although new chemotherapeutic agents have been developed, they still face many hurdles such as poor response rates, adverse events (AEs), and high overall costs. Although the benefits of locoregional versus systemic therapy remain controversial, locoregional therapy before systemic therapy is believed to be most effective for improving overall survival among non-surgically eligible patients [3].

Recent studies suggest that the combination of TACE and thermal ablation therapy is more effective than thermal ablation therapy alone [4–6]. However, these studies included patients with small (~ 3–5 cm), single tumours, whereas few studies have compared combination therapy versus TACE alone for intermediate-stage disease [7]. Also, the application of thermal ablation for large or multi-nodular HCC is technically difficult, as these types of tumours are prone to recur. Accordingly, few studies have evaluated local treatment for advanced-stage HCC. We postulate that combination of TACE and radiofrequency ablation (RFA) therapy could improve the rate of complete response (CR) and confer a survival benefit among patients with intermediate but beyond-the-Milan criteria. Although thermal ablation may not be applicable to large HCC, viable tumours after TACE are usually smaller than naïve tumours; thus, thermal ablation may also be applicable for viable tumours. The purpose of this trial is to compare 1-month tumour responses between patients with beyond-the-Milan HCC who receive combination TACE and RFA therapy versus TACE monotherapy.

### Objectives {7}

The primary endpoint is 1-month CR in patients with beyond-the-Milan criteria HCC who receive combination TACE and RFA therapy versus TACE monotherapy.

The secondary endpoints are:

1. Progression-free survival duration
2. Objective response rate (CR + partial response)
3. Number of treatments until CR
4. Overall survival rates (6, 12, and 24 months)
5. Change in liver function (i.e., Child–Pugh score) within 6 months

### Trial design {8}

This trial is a prospective, randomized, controlled, investigator-initiated, multi-institutional, open-blind, superiority study conducted in three academic hospitals of The Catholic University of Korea. Patient allocation will be performed with a 1:1 ratio for two parallel groups using computerized randomization.

## Methods: participants, interventions, and outcomes

### Study setting {9}

**Fig. 1:**
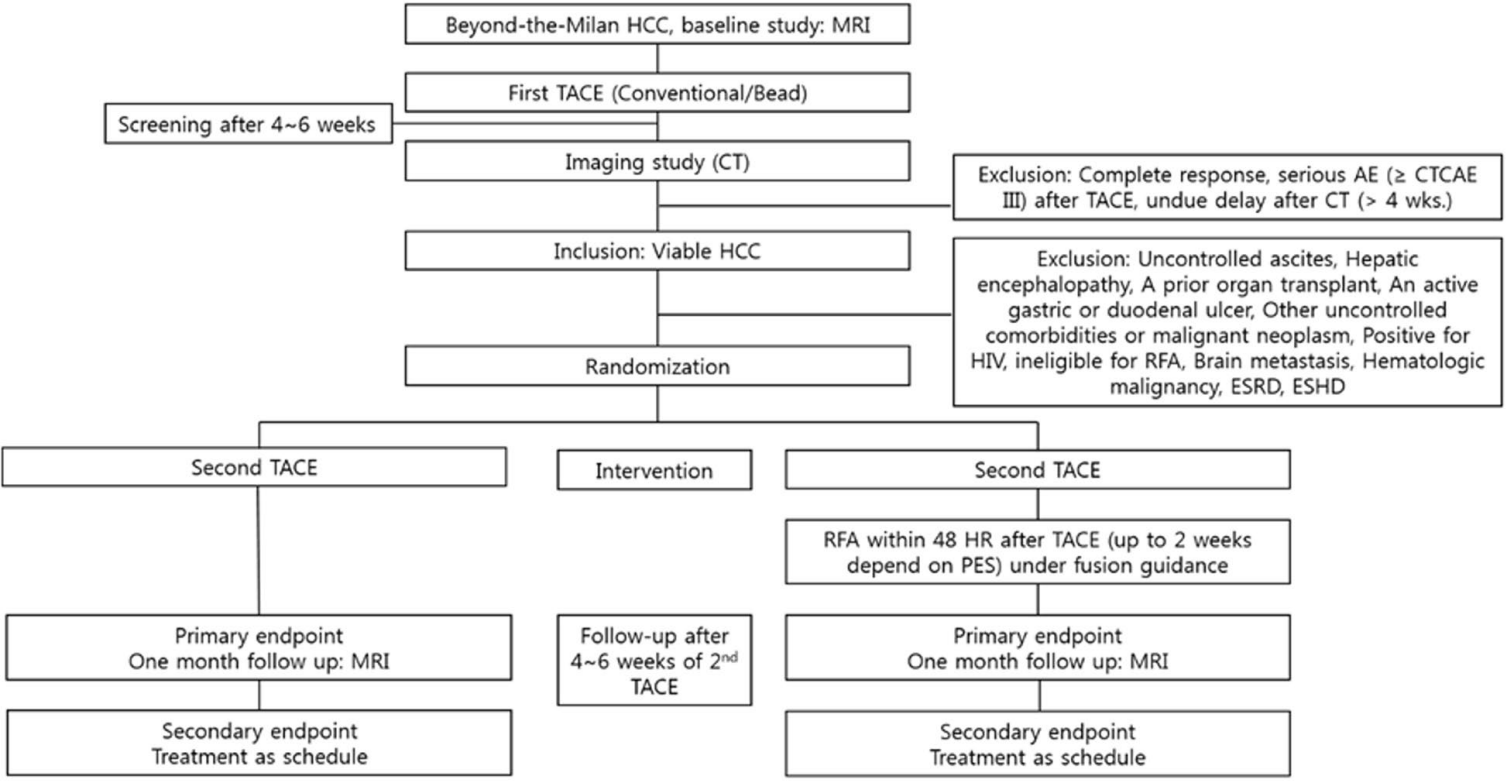
CONSORT flow chart

This multi-institutional randomized controlled trial will be conducted in three hospitals of The Catholic University of Korea: Seoul St. Mary’s Hospital, Eunpyeong St. Mary’s Hospital, and Incheon St. Mary’s Hospital. The study design is depicted in Fig. 1.

### Eligibility criteria {10}

#### Inclusion criteria

1. Patients ≥ 19 years of age with an established diagnosis of HCC by radiologic study in high-risk patient groups (e.g., hepatitis B or C, alcoholism, autoimmune liver disease) or pathologic examination
2. Single nodular HCC with a size of 5–8 cm
3. Dual nodular HCC with a maximal size of ≤ 8 cm
4. Triple nodular HCC with a maximal size of ≤ 5 cm
5. Patients meeting criteria (2) or (3) with viable HCC after first TACE^a^
6. Child–Pugh score of ≤ 8
7. No radiologic evidence of tumour invasion into the portal vein
8. No metastatic lesions outside the liver
9. No pathologic hematologic or renal functional abnormalities^b^
10.  Eastern Cooperative Oncology Group performance score of 0 or 1

a Viable HCC will be evaluated within 4–6 weeks after first TACE. Viable HCC should be treated within 4 weeks of radiologic evaluation after first TACE. Patients with longer treatment delays will be excluded from trial enrolment
b Haemoglobin > 8.5 g/dL, absolute neutrophil count > 750/µL, platelets > 30,000/µL, international normalized ratio ≤ 1.5, and serum creatinine level ≤ 1.5 mg/dL.

#### Exclusion criteria

1. Patients with grade ≥ 3 AEs according to the Common Terminology Criteria for Adverse Events (CTCAE) after first TACE
2. Unfeasible location of viable HCC for RFA
3. Uncontrolled ascites
4. Uncontrolled hepatic encephalopathy
5. Recent variceal bleeding within 6 months
6. Prior organ transplant
7. Active gastric or duodenal ulcer
8. Other uncontrolled comorbidities or malignant neoplasms (except for tumours with CR more than 5 years prior)
9. Positive for human immunodeficiency virus
10.  Hematologic malignancy
11.  End-stage renal disease: patients not in a haemodialysis state (eGFR < 30 mL/min). Ongoing haemodialysis patients will be eligible for trial enrolment
12.  End-stage heart failure (New York Heart Association stage 3 or 4 or history of myocardial infarction within prior 12 months)
13.  Brain metastasis

### Who will take informed consent? {26a}

Informed consent will be obtained by investigators (i.e., hepatologists) in the outpatient clinic at each hospital after the first TACE. A detailed explanation of the trial and information sheets will be given to patients before obtaining their informed consent. No identifiable personal data will be published.

### Additional consent provisions for collection and use of participant data and biological specimens {26b}

We will request consent for review of participants’ medical records, and for the collection of blood samples to assess tumour markers (primary outcome) and liver function tests (secondary outcome). There will be no additional consent for ancillary studies.

### Interventions

#### Explanation for the choice of comparators {6b}

We hypothesize that tumour response will differ between patients with viable beyond-the-Milan HCC who receive combination TACE and RFA therapy versus TACE monotherapy.

### Intervention description {11a}

#### Before allocation

Patient eligibility will be evaluated after the first TACE. Patients with viable HCC after first TACE will be included in the trial. First TACE will be performed with ethiodized oil or drug-eluted beads with adriamycin (50–100 mg depending on tumour volume or liver function). The selection of ethiodized oil versus drug-eluted beads will be determined by discussion among physicians and interventional radiologists. All feeding arteries will be treated via superselective catheterization. Gelfoam particles will be used with ethiodized oil as is conventional for TACE.

#### Allocation

One month after first TACE, patients with viable tumour as revealed by computed tomography (CT) will be randomly allocated into combination therapy or monotherapy groups on the morning of the day of second TACE.

#### After allocation

All patients will undergo second TACE for the viable portion of the HCC with ethiodized oil and Gelfoam particles. Interventional radiologists performing second TACE will be blinded to patient allocation, and TACE will be performed in a conventional manner.

Patients in the combination TACE and RFA therapy group will be treated with additional RFA of the viable portion of the tumour under the guidance of fusion images from ultrasound and CT. RFA will be implemented within 1–2 days after second TACE. Whether RFA can be delayed for up to 1 month will depend on the medical condition of the patients. RFA will be performed by physicians at the three hospitals with more than 8 years of experience. A 17-gauge radiofrequency electrode system including three electrodes with a length of 150 mm and a 30-mm ablation zone (Octopus; STARmed, Goyang-si, Korea) will be used. The number of electrodes used will be determined based on the size of the viable tumour and the physicians’ discretion. However, physicians will attempt to obtain an ablation margin of at least a 1-cm perimeter around the viable tumour.

Patients in the TACE monotherapy group will be discharged from the hospital without further intervention if they experience no AEs.

#### Follow-up and primary endpoint evaluation

All patients will be followed for 4–6 weeks after admission for second TACE on an outpatient basis. Magnetic resonance imaging (MRI) with gadoxetic acid (Primovist; Beyel-Shering Pharm, Beyel, Germany) and laboratory tests, including tumour markers and a liver battery, will be performed. Tumour response according to modified Response Evaluation Criteria in Solid Tumours (mRECIST) criteria will be assessed by two independent radiologists blinded to patient allocation (Asan Imaging Metrics, Seoul, Korea). In the case of disagreement, a third independent radiologist will review the imaging results and make a final decision.

#### Criteria for discontinuing or modifying allocated interventions {11b}

Patients who are assigned to the combination therapy group and do not receive RFA within 2 days after second TACE without consent of the investigator will be excluded. However, RFA can be delayed up to 1 month for patients with severe post-embolization syndrome depending on the hepatologist’s discretion.

#### Strategies to improve adherence to interventions {11c}

To prevent loss of follow-up between combination therapies, both second TACE and RFA will be performed during a single hospital admission unless the patient is unable to undergo RFA due to severe postembolization syndrome.

#### Relevant concomitant care permitted or prohibited during the trial {11d}

According to the nationwide health insurance system, additional use of systemic chemotherapy is not reimbursed during locoregional treatment and its evaluation period (4–6 weeks). Although external radiation can be applied in patients with malignant portal vein thrombosis, this study will not include patients with portal vein thrombosis. Therefore, there is little chance of concomitant care during the intervention and evaluation period. After evaluation of the primary endpoint, other systemic or locoregional treatments can be applied according to patients’ disease status.

#### Provisions for post-trial care {30}

During the intervention period, patients will be closely monitored by investigators. Regular vital signs and biochemical laboratory tests will be performed. After the intervention and evaluation period, patients will undergo a conventional treatment course. Investigators will attempt to attenuate any damage from the interventions. When it is impossible to recover from irreversible injury, an insurance program associated with this trial will provide compensation. Patients will not be compensated for their participation in this trial. After evaluating primary endpoint, patients will be followed by their own treatment plan (local or systemic therapy) based on disease status.

#### Outcomes {12}

The primary endpoint is 4–6-week CR in patients with viable tumour after first TACE. Tumour response rate will be evaluated in accordance with mRECIST [8]. In patients with multiple tumours, the sum of the largest diameters of viable lesions will be calculated.

Secondary outcomes include the following: (1) progression-free survival, defined as the time between random allocation and first progression; (2) overall response rate, defined as CR + partial response; (3) number of treatments (i.e., TACE) until achieving CR; (4) overall survival rate (i.e., 6, 12, and 24 months) and cause of death; and (5) change in liver function within 6 months based on liver function tests and Child–Pugh score.

#### Participant timeline {13}

**Table:** 

	**Study period**							
	**Enrolment**	**Allocation**	**Post-allocation**					**Close-out**
Timepoint**	***-1 to -2 w***	**0**	***2 w***	***1 m***	***2 m***	***6 m***	***12 m***	***24 m***
**Enrolment:**								
**Eligibility screen**	X							
**Informed consent**	X							
**Laboratory tests**	X							
**Allocation**		X						
**Interventions:**								
**TACE + RFA**			X					
**TACE**			X					
**Assessments:**								
**Baseline MRI**	X							
**CT after first TACE**		X						
**Follow-up MRI**				X				
**Regular follow-up (lab, CT or MRI)**					X	X	X	X

#### Sample size {14}

A previous study reports that the rate of CR after combination TACE and RFA therapy was 53% for patients with HCC tumours > 5 cm [9]. By contrast, the CR rate after TACE monotherapy was 26% for patients with intermediate-stage HCC [10]. Thus, we assume that the difference in CR rate between groups may be 28%. In this trial, we calculated that 48 patients per group are needed to achieve a two-sided α level of 0.05 and statistical power of 80%. A total of 120 patients will be included in this trial (i.e., 60 patients per group) to account for a 20% dropout rate (PASS, version 16, NCSS statistical software). Data will be analysed on an intention-to-treat basis according to patients’ originally assigned group.

#### Recruitment {15}

Patients with beyond-the-Milan criteria HCC will be considered as candidates for trial enrolment in concordance with the inclusion and exclusion criteria. Written informed consent will be obtained from each patient. A total of 120 patients will be enrolled from the three hospitals by competitive recruitment.

### Assignment of interventions: allocation

#### Sequence generation {16a}

An independent statistician will generate random numbers using STATA (ver. 16, StataCorp LLC) using a 1:2 to 1:6 random block stratified by three strata (i.e., hospitals). Data will be stored in a secure online database (i.e., REDCap) using electronic case report forms (e-CRFs).

#### Concealment mechanism {16b}

Informed consent will be obtained at the hepatology outpatient clinic. After agreeing to participate in the trial, patients will be admitted to the hospital based on its interventional radiology schedule. Random allocation will be performed on the day of second TACE. An outpatient hepatology clinic nurse who is independent from this trial will access the secure, password-protected, online database (i.e., REDCap) to perform patient allocation and notify the hepatologist only. All researchers and patients will be blinded to allocation status except for the hepatologist. Patient allocation will be released after the completion of second TACE.

#### Implementation {16c}

An independent statistician (YJY) will generate a stratified computer-generated block randomization list using STATA. A hepatologist will screen patients on an outpatient basis. If a patient agrees to participate in the trial, then the hepatologist will register the patient in the online database. Patients will be admitted to the hospital the day before second TACE. Second TACE will be performed by a dedicated interventional radiologist. After second TACE, patient allocation will be opened. According to their allocation, patients will be subsequently treated with RFA or discharged from the hospital.

### Assignment of interventions: blinding

#### Who will be blinded {17a}

The interventional radiologist and patients will be blinded to patient allocation before second TACE and unblinded after second TACE. The independent outcome assessors will be blinded to patient allocation. The primary outcome (i.e., CR) will be assessed by comparing initial and follow-up MRIs.

#### Procedure for unblinding if needed {17b}

After allocation, patients and the interventional radiologist performing second TACE will be blinded. After second TACE, the hepatologist will reveal patient allocation.

### Data collection and management

#### Plans for assessment and collection of outcomes {18a}

Consistent with the usual treatment course for HCC, initial laboratory tests and MRI will be performed. Tumour marker assessment (i.e., alpha-fetoprotein and protein induced by vitamin K absence-II) and MRI will also be performed 1 month after treatment. Blood laboratory tests and CTs will be performed depending on patients’ medical conditions as decided by the physician.

#### Plans to promote participant retention and complete follow-up {18b}

Patients will receive a recommendation to continue follow-up care at their treating hospital. There are no plans to promote participation retention.

#### Data management {19}

All data will be stored securely in an online database (i.e., REDCap). The investigators will be responsible for data registration and management. Data will be checked by at least two investigators.

#### Confidentiality {27}

Data from each patient will be assigned a code number, which will be the only link to patients’ identities during the trial period to maintain anonymity. Signed informed consent will be secured in a locked cabinet in a secure place in each hospital. Data will be stored for 3 years after trial completion according to the Enforcement Decree of the Bioethics and Safety Act of Korea. Data will be destroyed after 3 years; however, data storage may be extended with institutional review board (IRB) permission.

#### Plans for collection, laboratory evaluation, and storage of biological specimens for genetic or molecular analysis in this trial/future use {33}

Baseline and follow-up procedural laboratory test results and MRI images will be stored. There is no future research plan.

### Statistical methods

#### Statistical methods for primary and secondary outcomes {20a}

Parametric data will be reported as mean and standard deviation. Non-parametric data will be reported as median and range. Independent sample *t*-tests or Mann–Whitney *U* tests for continuous variables and chi-square or Fisher’s exact tests for categorical variables will be used to compare treatment groups. For the primary endpoint, rate of CR will be compared using a chi-square test. For secondary endpoints, Kaplan–Meier survival analysis and log-rank tests will be performed. A *p*-value < 0.05 will be considered statistically significant.

#### Methods for additional analyses (e.g., subgroup analyses) {20b}

Patient subgroups will be analysed according to treating hospital and liver function (i.e., Child–Pugh score).

#### Methods in analysis to handle protocol non-adherence and any statistical methods to handle missing data {20c}

Data produced following protocol non-adherence will not be included in the trial and will be disclosed. All researchers will make an effort to reduce missing data to a minimum. We will handle missing data with multiple imputation (MICE Package, R ver. 4.0.3, The R Foundation for Statistical Computing, Vienna, Austria). Missing values will be handled appropriately following established guidelines [11].

#### Plans to give access to the full protocol, participant level-data, and statistical code {31c}

The full protocol will be available on the registry website and is published here.

### Oversight and monitoring

#### Composition of the coordinating centre and trial steering committee {5d}

JHK and DJS will take full responsibility for scientific validity, study quality, study conduct, procedures, patient management after AEs, and the quality of the final trial results and reports. All investigators will share information through periodic meetings and will discuss appropriate trial management when problems occur. This study will be conducted without set up of Trial Steering Committee or Stakeholder and Public Involvement Group.

#### Composition of the data monitoring committee, its role, and reporting structure {21a}

A data monitoring committee (DMC) will be constituted by three independent medical researchers (Yun-Jung Yang; Institute of Biomedical Science, Catholic Kwandong University International St. Mary’s Hospital, Incheon, Korea, Jaesin Lee; Department of Internal Medicine, Incheon St. Mary’s Hospital, The Catholic University of Korea, Incheon, Korea, Il Jung Kim; Department of Radiology, Bucheon St. Mary’s Hospital, College of Medicine, The Catholic University of Korea, Seoul, Korea). The DMC will check the appropriateness of source documents, patient compliance, protocol violations, the process of consent acquisition and storage, review of e-CRFs and investigators’ binders, collection of information on AEs, and follow-up procedures. The committee will be responsible for protecting participant safety, evaluating trial effectiveness, making decisions on trial conduct or cessation, and advising on protocol improvement.

Any procedure-associated AEs will be checked during the follow-up period and recorded in the e-CRFs. In the case of serious AEs (grade ≥ 3), AEs will be reported to the principal investigator (DJS) within 24 h. Each DMC member will judge whether the AE is associated with the investigated therapy, and any related serious AEs will immediately be reported to the IRB and principal investigator. Each researcher should report any serious AEs or other unexpected problems to the IRB within 15 working days. The DMC can recommend cessation of the trial if one treatment group has significantly more AEs than the other treatment group, the occurrence of serious AEs including procedure-related death, or complete enrolment of participants earlier than expected.

#### Adverse event reporting and harms {22}

AEs will be classified according to CTCAE [12]. Any serious AE (i.e., grade ≥ 3) will be reported to the IRB. Daily checks for each patient will be conducted by the research team at each hospital and communicated with the other research teams.

#### Frequency and plans for auditing trial conduct {23}

Patient monitoring by an independent monitor will take place until data from 40 participants are collected or every 12 months if 40 participants are not enrolled. The inspection centre of each hospital will designate inspectors to conduct systematic inspections of trial-related activities and documents. Evaluation of patients and data will be completely independent from the investigators at each hospital and the trial sponsors.

#### Interim analyses {21b}

No interim analyses are planned.

#### Plans for communicating important protocol amendments to relevant parties (e.g., trial participants, ethical committees) {25}

Any change to this trial protocol will be reported to the IRB of each hospital and trial registry.

#### Dissemination plans {31a}

The results of this trial will be published in a peer-reviewed medical journal.

## Discussion

In this trial, we will assess the efficacy of combination TACE and RFA therapy versus TACE monotherapy for patients with viable beyond-the-Milan criteria HCC after first TACE. Treatment of beyond-the-Milan criteria HCC still poses a great clinical challenge [13]. Barcelona Clinic Liver Cancer guidelines suggest that intermediate-stage HCC can be treated with TACE and that advanced-stage (portal invasion, N1, or M1) patients can be treated with sorafenib [2]. For beyond-the-Milan criteria patients, TACE alone is often not effective, as tumours tend to have multiple feeders, and a high tumour burden demands a large amount of embolic material, which can lead to incomplete treatment due to a risk of hepatic failure. Although sorafenib and new-generation systemic chemotherapies are reserved for patients with more advanced-stage disease, further improvements in response rates may not be expected [14]. Thus, it seems necessary to establish a more stratified treatment strategy for patients with intermediate but beyond-the-Milan criteria HCC.

Recent studies on combination TACE and RFA therapy for early- and intermediate-stage HCC demonstrate promising results compared with TACE or RFA alone [4, 6, 7, 15–17]. Theoretically, this combination therapy can overcome the limitations of each individual treatment. First, TACE can reduce tumour burden. Second, ablation could be more effective without heat sinking by decreasing arterial flow through embolization. Third, TACE can cover undetectable satellite nodules that can be missed by RFA [17]. In addition, lipiodol-uptake nodules after TACE can be precisely localized during RFA in the treatment of non-ultrasound-discernible and inaccessibly located HCC [18]. Whereas most studies on combination therapy concerned tumours sized < 5 cm, it is unclear whether combination therapy could also be beneficial for beyond-the-Milan criteria patients.

Treatment of beyond-the-Milan criteria HCC is clinically challenging due to the size and number of tumours and the possibility of metastasis and vascular invasion. Patients with beyond-the-Milan criteria HCC have not been candidates for ablation therapy, and guidelines instead recommend sorafenib treatment. However, local treatments such as TACE applied before systemic treatment have been attempted because some patients with intermediate-stage disease show better responses to localized treatment and can succeed in downstaging compared with patients receiving systemic treatment. However, TACE with tumours > 5 cm often leaves a viable portion of tumour requiring repeated treatment [9]. MRI ~ 1 month after TACE can detect the viable portion of HCC, which can be smaller than its initial size and possibly treatable by RFA.

This trial protocol has some limitations. First, this is an open-blind study, although patients will be blinded up to their completion of second TACE. Second, the mechanism by which combination therapy may confer a survival benefit is unclear, and the magnitude of the survival benefit compared with that following monotherapy may be more limited than expected. Third, advanced-stage tumours have a high possibility of vascular invasion that can fail to be revealed by imaging, leading to worse prognosis than expected in these patients.

In summary, this trial will evaluate the efficacy and safety of combination TACE and RFA therapy versus TACE monotherapy among patients with viable beyond-the-Milan criteria HCC after first TACE.

## Trial status

This trial is currently recruiting participants. Initial recruitment started on 16 November 2020. Recruitment and patient follow-up are ongoing. The approximate date of recruitment completion is June 2026. This protocol is version 2.1, dated 1 February 2023.

## Data Availability

Datasets generated by and/or analysed in this trial will be available from the corresponding author on reasonable request after all identifiable information has been removed.

## Abbreviations

AE: Adverse event
CT: Computed tomography
CTCAE: Common Terminology Criteria for Adverse Events
DMC: Data monitoring committee
e-CRF: Electronic case report form
HCC: Hepatocellular carcinoma
mRECIST: Modified Response Evaluation Criteria in Solid Tumours
MRI: Magnetic resonance imaging
RFA: Radiofrequency ablation
TACE: Transcatheter arterial chemoembolization
